# Cues to deception: can complications, common knowledge details, and self-handicapping strategies discriminate between truths, embedded lies and outright lies in an Italian-speaking sample?

**DOI:** 10.3389/fpsyg.2023.1128194

**Published:** 2023-04-26

**Authors:** Letizia Caso, Lucrezia Cavagnis, Aldert Vrij, Nicola Palena

**Affiliations:** ^1^Department of Human Sciences, Libera Università Maria SS. Assunta, Rome, Lazio, Italy; ^2^Department of Human and Social Sciences, University of Bergamo, Bergamo, Lombardy, Italy; ^3^Department of Psychology, University of Portsmouth,, Portsmouth, United Kingdom

**Keywords:** complications, common-knowledge details, self-handicapping strategies, lying, verbal cues to deception

## Abstract

Deception research has shown that analysing verbal content can be effective to distinguish between truths and lies. However, most verbal cues are cues to truthfulness (truth tellers report the cue more than lie tellers), whereas cues to deception (lie tellers report the cue more than truth tellers) are largely absent. The complication approach, measuring complications (cue to truthfulness), common knowledge details (cue to deception), self-handicapping strategies (cue to deception), and the ratio of complications, aims to fill this gap in the literature. The present experiment examined the effectiveness of the complication approach when varying the amount of lying, with an Italian sample. Seventy-eight participants were assigned to one of three different experimental conditions: Truth tellers (telling the truth about the event), embedders (providing a mixture of truthful and false information) and outright lie tellers (providing false information). Participants were interviewed about a past experience concerning an out of the ordinary event. Complications discriminated truth tellers from lie tellers. The absence of significant effects for common knowledge details and self-handicapping strategies, the limitations of the experiment and suggestions for future research are discussed.

## Introduction

Scholars working in the area of investigative interviewing and lie detection have spent over five decades searching for nonverbal and verbal cues to deception (Vrij, 2008; Vrij et al., 2022b). Initially, the focus was on nonverbal cues (Ekman and Friesen, 1969; Zuckerman et al., 1981; Ekman, 2001; Bond et al., 2015). However, body language and facial expressions cues have shown to be unreliable cues to deception (DePaulo et al., 2003; Vrij, 2008; Burgoon, 2018). Scholars have therefore suggested to explore verbal content.

One of the first tools to analyse verbal content was Statement Validity Assessment (SVA), which assumes that truths are qualitatively different from lies (Undeutsch, 1967). SVA comprises instructions on how to conduct an interview and two checklists: the Criteria Based Content Analysis (CBCA) and a Validity Checklist. The former includes 19 verbal criteria that are thought to be more frequently present in truthful than in deceptive statements. The Validity Checklist examines whether the CBCA scores could have been influenced by factors other than veracity (Vrij, 2005, 2015). Reality Monitoring is another verbal veracity assessment tool. It builds on memory research (Johnson and Raye, 1981) and includes eight verbal criteria. Meta-analytic work showed that both tools can discriminate truth telling from lying with an accuracy rate of up to 70% (Hauch et al., 2017). The verifiability approach (VA) is a more recently developed tool (VA, Nahari et al., 2014). It focuses on details that can be potentially verified by investigators, including activities (i) carried out with or (ii) witnessed by named persons, (iii) captured on CCTV cameras or (iv) leaving a trace (receipts, debit card use, phone calls). A meta-analytic approach also provides support for the VA (truth tellers report more verifiable details than lie tellers), especially when interviewees are informed that the investigator may check their details (Palena et al., 2021b).

Although the available literature shows that verbal content analysis can be used for lie detection purposes, most verbal criteria included in the tools are indicative of truth telling (truth tellers provide a cue more than lie tellers). Cues indicative of lying (lie tellers provide a cue more than truth tellers) are rare (Nahari et al., 2019). The exception is the cognitive operations cue which is part of Reality Monitoring. However, that cue does not discriminate truth tellers from lie tellers (Gancedo et al., 2021). Furthermore, it is important to examine a mixture of cues to truthfulness and deceit as this will allow making verbal lie detection tools, which are mostly focused on truth cues, more attractive for practitioners, who usually look for signs of deception (Vrij et al., 2022a). The distinction between cues to truthfulness and cues to deception is important. Although one might believe that they are the same and that they work equally well for both truth detection and lie detection, this is not the case. Research shows that a lot of detail can be interpreted as a cue to truthfulness, but lack of detail does not imply deception *per se*. For example, a truth teller who can provide a CCTV recording as evidence for their statement can demonstrate their honesty. However, another truth teller who cannot provide such evidence is still telling the truth even without such evidence.

The term “detail” refers to the total amount of detail in a statement, regardless of the specific types of detail being considered. However, total details can also be broken down in more specific types of detail, such as perceptual details (information related to the five human senses) and spatial details (information concerning places and spatial arrangements of objects, people, etc.). In an effort to deal with the lack of lie cues, Vrij and colleagues recently introduced a mixture of three specific cues to truthfulness and deceit (Vrij et al., 2018a, 2021; Vrij and Vrij, 2020): Complications (cue of truthfulness) and common knowledge details and self-handicapping strategies (cues to deceit). They also considered the proportion of complication to the sum of complications, common knowledge details and self-handicapping strategies (a ratio score). Complications are pieces of information that make the interviewee’s statement more complicated (e.g., “We flew from Rome to New York *via* Philadelphia because we have some friends living in Philadelphia”). Complications are also considered in CBCA coding. However, in CBCA complications need to be unexpected, which is not the case in Vrij et al. (2021) approach. Truth tellers report more complications than lie tellers because lie tellers try to keep their stories simple. Common knowledge details are pieces of information related to scripts or stereotypical mentionings of well-known situations (e.g., “The first day in Paris we visited the Louvre Museum where we saw the Mona Lisa”). When truth tellers report an experience, they often include some unique personal experiences in their accounts. Lie tellers, who lack such personal experiences, are tempted to draw on general knowledge (Vrij et al., 2018a). The self-handicapping strategies cue refers to justifications that people use when they cannot provide information (“There is not much to say about this bungee jump, it all happened very quickly”). Reporting self-handicapping strategies offers lie tellers an excuse not to provide information. The complication ratio is defined as (complications/[complications + common knowledge details + self-handicapping strategies]). A recent meta-analysis of the complication approach (Vrij et al., 2021) showed that truth tellers reported more complications (*d* = 0.51 to *d* = 0.62) and fewer common knowledge details (*d* = −0.40 to *d* = −0.46) and fewer self-handicapping strategies (*d* = −0.37 to *d* = −0.50) than lie tellers. The complication ratio variable was not included in the meta-analysis.

### Lying strategies

There are different ways in which people can lie, including by telling total falsehoods (i.e., making up stories entirely by reporting invented information) or by telling embedded lies (including false information in an otherwise truthful story). Embedded lying could involve telling the truth about one part of the day (for example the morning) and lying about another part of the day (for example the afternoon). Vrij and Mann (2001) found that a convicted murderer did exactly this. Palena et al. (2019) developed an experimental design where participants were asked to tell the truth for one part of the story but to lie about the other part.

Research has shown that most people tend to tell embedded lies (for a more detailed discussion of lying strategies, see for example Weiss and Feldman, 2006; Leins et al., 2012; Nahari and Nisin, 2019; Orthey et al., 2019; Verigin et al., 2019). In deception research lie tellers sometimes tell total falsehoods and sometimes embedded lies but we are not aware of research that compares these two ways of lying. However, such a comparison is important. It sounds plausible that cues to truthfulness and deception covary with the degree of lying with fewer cues to truthfulness and more cues to deception arising in the more extreme form of lying (telling total falsehoods).

The complication approach has been tested in various countries, including the United Kingdom, United States, Russia, South Korea, Mexico and Lebanon (Vrij et al., 2018b, 2019a,b). The complication approach obtained general support in these different countries but was never examined in Italy. Although we expected the findings in an Italian sample not to differ from other samples, we felt it important to conduct an experiment in Italy. Practitioners typically prefer that a lie detection tool is tested in their own country before considering using the tool.

Building on the available literature, we hypothesised that truth tellers would report more complications (*H1*a) and a higher proportion of complications (*H2*a), but fewer common knowledge details (*H3*a) and fewer self-handicapping strategies (*H4*a) than those participants who were requested to tell an embedded lie who in turn were expected to report more complications (*H1*b) and a higher proportion of complications (*H2*b) but less common-knowledge details (*H3*b) and less self-handicapping strategies (*H4*b) than those participants who were asked to tell an outright lie.

## Methods

### Participants

An *a-priori* sample size calculation conducted in GPower 3.1 (Faul et al., 2007), with *F* as the test family, an effect size set at *f* = 0.40 (Vrij et al., 2021), α set at 0.05 and power at 0.80 indicated that at least 66 participants were required for the experiment. In total, 78 participants took part in the experiment and all university students were recruited during university lectures and with flyers. Sixty-seven (86%) identified themselves as females, the remaining identified themselves as male. Age ranged from 20 to 60 years old (*M* = 23.53, *SD* = 6.20).

### Procedure

A list of potential participants was obtained during university lectures. Volunteers were emailed instructions about the experiment 2 days before the interview. This reflects police practice in Italy, where interviewees are informed in advance that they will be interviewed by the police. Participants were told that they had to recount a memorable, out of the ordinary, event that happened within the last 12 months, building on Vrij et al. (2017) procedure. They also had to provide a title for the event to be used by the interviewer in the upcoming interview.

Our procedure was not identical to that used by Vrij et al. (2017). First, when describing the event they chose, one third of the participants were asked to tell the truth about the entire event (referred to as “truth tellers”), one third of them was asked to lie about the entire event (referred to as “outright lie tellers”) and one third of them was asked to tell the truth about half of the day but to lie about the other half (referred to as “embedders”). Concerning this embedders group, half of the participants were asked to tell the truth about what happened before midday but to lie about what happened after midday, whereas the other half was asked the opposite. In this way we counterbalanced the truth and lie parts of the story. Second, in Vrij et al. procedure, lie tellers’ stories were matched to truth tellers’ stories (i.e., lie tellers were asked to invent a story about a truth teller’s event). Instead, in our procedure, we asked lie tellers to report false information about their own suggested stories. We decided to do so as one of the aims of the present experiment was to mirror real-life situations where interviewees base their lies on their own experiences.

The instructions also informed participants that they could earn one additional point for a university exam if they would be believed by the interviewer. In contrast, if the interviewer would not believe them, they would have to write a statement concerning why, in their opinion, the interviewer did not believe them. In reality, all participants were offered the university exam point, and nobody was asked to write the statement.

On the day of the interview, the participants first read and signed the consent form. They were then brought to the interview room where they met the interviewer. The interviews started with the interviewer saying: “*I am aware that on day X you* (title of the event). *Could you please describe this event in as much detail as possible, from its beginning to its end, that is, from when you woke up to when you went to sleep?*” Once the participant stopped talking, they were asked *“Could you please now describe in as much detail as possible what happened on day X when (title of the event), this time focusing only on what happened in the morning, that is from when you woke up to midday?”* Once the participant stopped talking, they were asked *“Could you please now describe in as much detail as possible what happened on day X when (title of the event), this time focusing only on what happened in the afternoon, that is from midday to when you went to sleep?”* Although we are aware that an event could have lasted less than an entire day, we decided to structure the questioning as above for two main reasons. First, as said above, to mirror real-life situations where an interviewee is questioned about what happened the day of the event under investigation (see for example Vrij and Mann, 2001). Second, to create the embedded lie condition.

Once the interview had finished, the participants were asked to fill in a questionnaire where all answers were provided on a 0% (not at all) to 100% (completely/very much) scale. The questions concerned the amount of lying (*“How much did you lie while reporting the event?”*), motivation (*“How motivated were you to be believed by the interviewer,”* and *“How motivated were you to report details?”*), difficulty of the interview (*“How difficult did you find the interview?”*), plausibility of having to write a statement as to why the interview did not believe them (*“How likely did you think it was that you have to write a statement about why the interviewer did not believe you?”*), memory (*“How would you rate your memory of the event?”*), preparation time (*“How much time did you spend preparing for the interview in the time between when you received the email about the experiment and when the interview took place?”*), preparation effort (*“How much effort did you put in the preparing for the interview in the time between when you received the email and when the interview took place?”*) and credibility (*“How credible do you think you were?”*). The experiment was conducted following the Declaration of Helsinki and the Ethical Guidelines for research provided by the Italian Psychological Association (Associazione Italiana di Psicologia, 2015).

### Coding

The interviews were transcribed and coded following Vrij et al. (2017) coding scheme of complications (e.g., *“I put the short-sleeves fur on, after which I realized that it was too cold”*; *“The 11 am train was delayed”*; *“As I walked into the store, I did not see a step. I stumbled and fell on the floor”*), common knowledge details (e.g., *“I went to Milan and visited several shops in the city centre”*; *“On my little sister’s birthday, we opened the presents for her”; “We went to New York and visited the Statue of Liberty”*), and self-handicapping strategies (e.g., *“I have nothing much to say about the robber, as it went very quickly”*; *“I cannot tell much as I fell asleep during the journey”*; *“I cannot remember as it happened a while ago”*).

The answers to the free recall and the two follow-up questions were coded. Two coders independently coded 100% of the transcripts. Each of the three types of detail was counted only once and repetitions were not considered. To assess inter-coder agreement, we calculated Intraclass-Correlation Coefficients (ICC) by using a two-way random, single measure, model (ICC 2, 1) (Shrout and Fleiss, 1979). ICC was of 0.91 for complications, 0.96 for common knowledge details and 0.99 for self-handicapping strategies, indicating high agreement between the two coders. We used the ratings of the most experienced coder in the analyses.

## Results

### Manipulation check

A manipulation check was conducted on the truth telling-lying manipulation and showed that outright lie tellers reported to have lied more than embedders, who in turn reported to have lied more than truth tellers (Table 1). This means that the veracity manipulation was successful.

**Table 1:** Manipulation checks and post-interview questionnaire statistics.

		M (SD)		
	*F*(2,75)	Truth tellers	Embedders	Outright lie tellers
Motivation to be believed	2.21	83.08 (17.61)	85.39 (17.49)	75.77 (16.53)
Motivation to be detailed	4.65*	87.31 (13.13)^a^	81.92 (13.86)^ab^	75.00 (16.55)^bc^
Perceived interview difficulty	8.03***	24.62 (24.04)^a^	46.15 (24.34)^b^	50.39 (26.15)^b^
Perceived probability of having to write a statement	12.08***	27.69 (29.16)^a^	55.00 (22.14)^b^	58.46 (22.22)^b^
Memory of the event	0.25	83.46 (16.23)	84.23 (12.39)	81.54 (13.47)
Preparation time	5.55**	30.77 (17.19)^a^	50.00 (23.83)^b^	44.23 (22.48)^ab^
Preparation effort	9.64***	42.31 (25.03)^a^	70.39 (16.61)^b^	53.50 (26.73)^a^
Amount of lying	149.84***	1.92 (4.02)^a^	47.69 (18.61)^b^	75.39 (18.81)^c^
Perceived credibility	14.30***	86.92 (7.36)^a^	73.08 (12.89)^b^	65.77 (20.23)^b^

Different superscripts indicate *p* < 0.05, *p <0.05, **p < 0.01, ***p < 0.001.

### Post-interview questionnaire analyses

Several ANOVAs and Tukey *post-hoc* tests were conducted on the post-interview questionnaire (Table 1). The experimental condition was not associated with the motivation to be believed nor with the memory for the event, but it was significantly associated with the motivation to be detailed, the perceived difficulty of the interview, the perceived likelihood of being requested to write a statement and preparation time and effort. Outright lie tellers were less motivated to be detailed than truth tellers, which supports the idea that lie tellers prefer to keep their stories simple (Verigin et al., 2019). Both embedders and outright lie tellers, compared to truth tellers, perceived the interview as more difficult (in alignment with the cognitive approach to deception, Vrij (2015) and thought it to be more likely to have to write a statement. The latter finding suggests that lie tellers thought that their lie would shine through, in accordance with the illusion of transparency theory (Gilovich et al., 1998).

Moreover, embedders reported to have spent more time than truth tellers to prepare for the interview. Embedders also reported to have put more effort in their preparation for the interview than truth tellers and outright lie tellers. Both embedders and outright lie tellers thought that they were less credible than truth tellers (Table 1).

**Table 2:** Descriptives of the dependent variables according to the veracity condition.

	M (SD) [95% CI]		
	Truth tellers	Embedders	Outright lie tellers
Complications	8.31 (5.58) [6.16, 10.45]	6.50 (3.90) [5.00, 8.00]	5.00 (4.02) [3.45, 6.55]
Common knowledge details	10.46 (4.94) [8.56, 12.36]	8.58 (5.15) [6.60, 10.56]	10.12 (7.66) [7.17, 13.06]
Self-handicapping strategies	0.27 (0.83) [−0.05, 0.59]	0.12 (0.43) [−0.05, 0.28]	0.23 (0.86) [−0.10, 0.56]
Complications ratio	0.43 (0.17) [0.36, 0.50]	0.43 (0.15) [0.37, 0.49]	0.33 (0.23) [0.24, 0.42]

### Hypothesis testing

Four ANOVAs were conducted, one for each dependent variable. Veracity (truth tellers vs. embedders vs. outright lie tellers) was the only factor. There was a significant effect for complications, *F*(2, 75) = 3.42, *p* < 0.05, *η*^2^ = 0.08. *Post-hoc* analyses showed that truth tellers (*M* = 8.31, *SD* = 5.58) reported more complications than outright lie tellers (*M* = 5.00, *SD* = 4.02), *t*(75) = 2.61, *p* < 0.05, *d* = 0.72. The number of complications in embedders’ statements (*M* = 6.50, *SD* = 3.90) did not differ from that in truth tellers’ statements, *t*(75) = 1.43, *p* = 0.33, *d* = 0.40, or from that in outright lie tellers’ statements, *t*(75) = 1.18, *p* = 0.47, *d* = 0.33. No significant differences occurred for common knowledge details, *F*(2, 75) = 0.71, *p* = 0.49, *η*^2^ = 0.02, self-handicapping strategies, *F*(2, 75) = 0.31, *p* = 0.73, *η^2^* = 0.01, and for the ratio of complications, *F*(2, 48.72) = 2.00, *p* = 0.15, *η*^2^ = 0.06 (Table 2). Taken together, the above results support the experimental hypothesis only for the variable complications when comparing truth tellers to outright lie tellers (*H1*).

## Discussion

In the present experiment, we examined the efficiency of the complication approach when comparing truth telling with embedded and outright lies. As predicted, we found the difference to be larger between truths and outright lies than between truth telling and embedded lies. However, only the number of complications was associated with veracity, with truth tellers reporting more complications than outright lie tellers.

The other three variables, common knowledge details, self-handicapping strategies, and the ratio of complications, were not associated with veracity. The nonsignificant results for common knowledge details and self-handicapping strategies can be interpreted in different ways. First, perhaps the instruction to think of and provide a statement concerning an out of the ordinary event impacted on the results. If an interviewee talks about an out of the ordinary event, lie tellers may find it inappropriate to report common knowledge details because they may think that sounds suspicious. Similarly, if the event is poor in verifiable details and/or sources (Nahari et al., 2014; Vrij et al., 2020), lie tellers perhaps think it is worth taking the risk to give extensive fabricated statements and do not think that self-handicapping strategies are required. Self-handicapping strategies are thought to be provided as justifications for not giving the required information to the interviewer (Vrij et al., 2021), but if an interviewer asks for more information to an interviewee concerning an event that is not checkable, it is possible that instead of using self-handicapping strategies to hide the true information the interviewee will substitute the “justification strategy” with a “providing unverifiable details” strategy.

Second, it is possible that the efficacy of the complication approach varies across people. Research has shown that there is high variability in deceptive communications due to interpersonal differences (Serota and Levine, 2015; Caso et al., 2018; Park et al., 2021). Building on this, scholars have made an effort to reduce the effect of interpersonal variability, for example by adopting specific interviewing strategies and within-subjects measures (Vrij, 2016; Vrij et al., 2018a; Verigin et al., 2020) but also by applying statical approaches that aims at grouping similar subjects (Palena and Caso, 2021; Palena et al., 2021a, 2022). It could be that people who score high on storytelling and on risk-taking and bluffing would provide more complications than people who score low on such variables, as the former would likely to be more apt and willing to create credible stories (storytelling skills) that include complication details that could be potentially proved wrong by an investigator (high risk-taking and bluffing tendency).

Embedders spent more time in preparing for the interview than truth tellers and they also put more effort in preparing for the interview than both truth tellers and outright lie tellers. It is not surprising that embedders prepared more than truth tellers, because lie tellers strategize more than truth tellers (Vrij, 2008). However, it was surprising that embedders put more effort in their preparations than outright lie tellers. Outright lie tellers would be expected to prepare more than embedders, as the former make-up their stories by reporting invented information. Hence, the act of creating a total falsehood is expected to require more fantasy, effort, creativity, and cognitive resources, thus, requiring more preparation effort. However, it could be that embedders had to put more effort in preparing their stories than outright lie tellers as the former needed to have their false information fit within their truthful part of the story in a consistent, coherent, and non-contradictory way.

Our experiment had some limitations. For example, we did not account for the effect of the topic of the statement provided by the interviewees. Moreover, we did not employ any within-subjects measure that could aid individual-case veracity decision. Further, we instructed participants to tell an embedded or outright lie. Hence, as is common practice in deception research, lying was not a participant’s choice, neither was the type of lie they told. The problem of letting participants decide for themselves to tell the truth or lie may result in confounded factors. For example, if most female participants decide to tell the truth, and most male participants decide to lie, veracity will be confounded with gender. However, we recognise that the use of instructed lies could be considered a limitation and suggest that future research accounts for unsanctioned lies (let participants decide themselves to tell the truth or lie). Last, inter-rater reliability for the coding of the statements was assessed and was high, but only the coding from one coder was used. Although this is common practice in lie detection research, not unitizing the coding from the two coders might be a limitation, as high agreement (and thus correlation) cannot exclude that the coders are coding different details. Future research should thus explore this aspect in more detail.

## Data availability statement

The raw data supporting the conclusions of this article will be made available by the authors, without undue reservation.

## Ethics statement

Ethical review and approval was not required for the study on human participants in accordance with the local legislation and institutional requirements. The patients/participants provided their written informed consent to participate in this study. The study was conducted in accordance with the Declaration of Helsinki and the guidelines provided by the Italian Psychological Association.

## Author contributions

LeC, LuC, AV, and NP contributed to the designing of the experiment and edited the first draft of the manuscript. LuC and NP conducted the analyses, interpreted the results, and wrote the first draft of the manuscript. All authors contributed to the article and approved the submitted version.

## Conflict of interest

The authors declare that the research was conducted in the absence of any commercial or financial relationships that could be construed as a potential conflict of interest.

## Publisher’s note

All claims expressed in this article are solely those of the authors and do not necessarily represent those of their affiliated organizations, or those of the publisher, the editors and the reviewers. Any product that may be evaluated in this article, or claim that may be made by its manufacturer, is not guaranteed or endorsed by the publisher.
